# Dr. Edwin A. Gaviller

**Published:** 1914-12

**Authors:** 


**Dr. Edwin A. Gaviller,** McGill 1873, died at his home in Hamilton, Ont., August 8, aged 74.

